# Electrophoretic mobility of supercoiled, catenated and knotted DNA molecules

**DOI:** 10.1093/nar/gku1255

**Published:** 2014-11-20

**Authors:** Jorge Cebrián, Maridian J. Kadomatsu-Hermosa, Alicia Castán, Víctor Martínez, Cristina Parra, María José Fernández-Nestosa, Christian Schaerer, María-Luisa Martínez-Robles, Pablo Hernández, Dora B. Krimer, Andrzej Stasiak, Jorge B. Schvartzman

**Affiliations:** 1 Department of Cellular and Molecular Biology, Centro de Investigaciones Biológicas (CSIC), Ramiro de Maeztu 9, 28040 Madrid, Spain; 2 Scientific and Applied Computing Laboratory, Polytechnic School, National University of Asunción, P.O. Box 2111, SL. San Lorenzo, Paraguay; 3 Center for Integrative Genomics, Faculty of Biology and Medicine, University of Lausanne, CH-1015 Lausanne, Switzerland

## Abstract

We systematically varied conditions of two-dimensional (2D) agarose gel electrophoresis to optimize separation of DNA topoisomers that differ either by the extent of knotting, the extent of catenation or the extent of supercoiling. To this aim we compared electrophoretic behavior of three different families of DNA topoisomers: (i) supercoiled DNA molecules, where supercoiling covered the range extending from covalently closed relaxed up to naturally supercoiled DNA molecules; (ii) postreplicative catenanes with catenation number increasing from 1 to ∼15, where both catenated rings were nicked; (iii) knotted but nicked DNA molecules with a naturally arising spectrum of knots. For better comparison, we studied topoisomer families where each member had the same total molecular mass. For knotted and supercoiled molecules, we analyzed dimeric plasmids whereas catenanes were composed of monomeric forms of the same plasmid. We observed that catenated, knotted and supercoiled families of topoisomers showed different reactions to changes of agarose concentration and voltage during electrophoresis. These differences permitted us to optimize conditions for their separation and shed light on physical characteristics of these different types of DNA topoisomers during electrophoresis.

## INTRODUCTION

**Figure 1.**
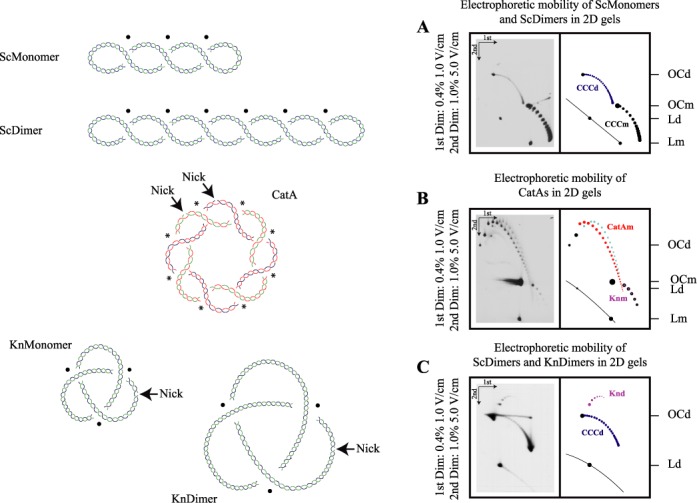
Cartoons and analysis of different topoisomers in two-dimensional (2D) agarose gels. A supercoiled monomer (ScMonomer), supercoiled dimer (ScDimer), nicked-catenated monomers (CatA), nicked-knotted monomer (KnMonomer) and nicked-knotted dimer (KnDimer) are represented. They all have the same mass and similar density of crossings (see text). Intramolecular crossings are marked with a black spot while intermolecular ones are pointed with an asterisk. The catenated rings in CatA are drawn in blue and red and in green and red to distinguish each of them. Immunograms of DNA samples enriched for different topoisomers analyzed by high-resolution 2D agarose gel electrophoresis are shown to the right: (**A**) DNA sample enriched for supercoiled monomers (CCCm) and dimers (CCCd). (**B**) DNA sample enriched for nicked-catenated monomers (CatAm) and knotted monomers (Knm). (**C**) DNA sample enriched for supercoiled dimers (CCCd) and knotted dimers (Knd). Electrophoresis conditions are detailed to the left of each immunogram. Interpretative diagrams are shown to the right. In these diagrams and the following figures supercoiled dimers (CCCd) are called ScDimers and are depicted in dark blue, nicked-casemated monomers (CatAm) are called CatAs and are depicted in red, and nicked-knotted dimers (Knd) are called KnDimers and are depicted in pink.

**Figure 2.**
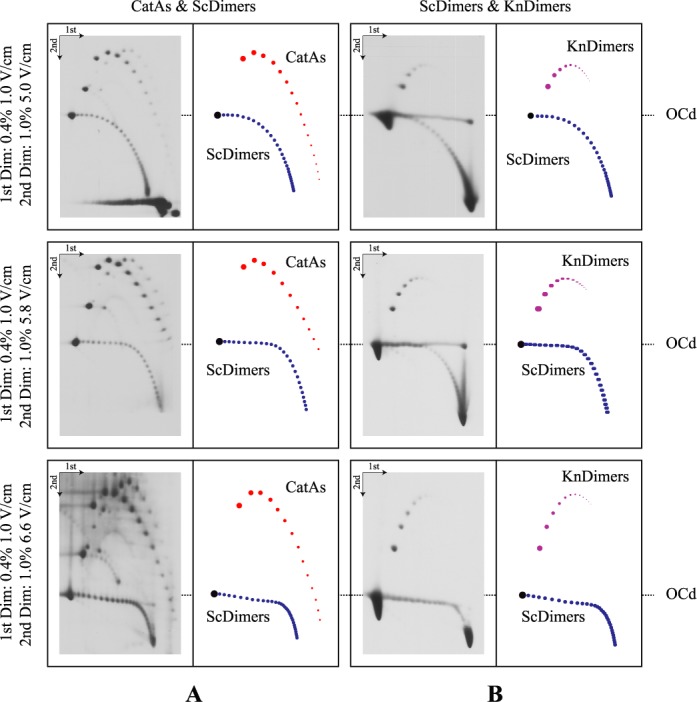
Immunograms of mixed DNA samples enriched for CatAs, ScDimers and KnDimers analyzed by 2D agarose gel electrophoresis where the second dimension was run in 1.0% agarose gels at different voltages. Electrophoresis conditions are detailed to the left and interpretative diagrams are shown to the right. OCd refers to the mobility of dimeric open circles used to align the different immunograms. CatAs are depicted in red, ScDimers in dark blue and KnDimers in pink.

**Figure 3.**
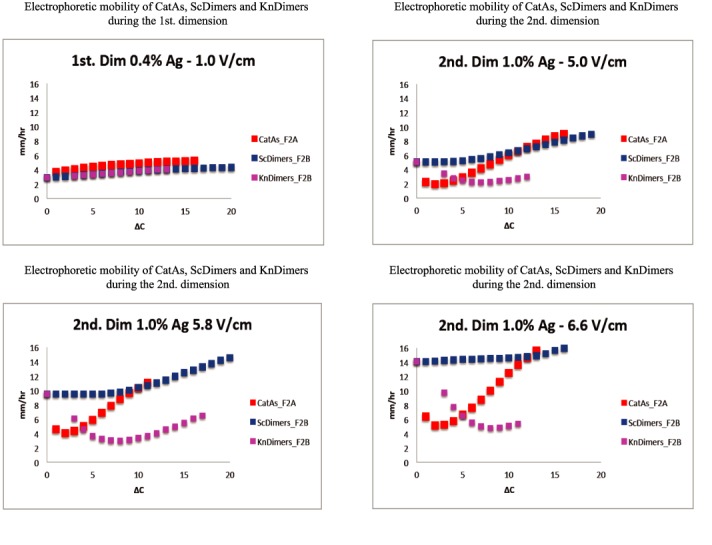
Comparison of the electrophoretic mobility of different topoisomers during the first and second dimension of 2D gels where the latter occurred at different voltages. Electrophoretic mobility as a function of Δ*C* is expressed in mm/h for the CatAs (depicted in red) shown in Figure 2A and for the ScDimers (depicted in dark blue) and KnDimers (depicted in pink) shown in Figure 2B. The actual numbers are shown in Supplementary Tables S1–S4).

**Figure 4.**
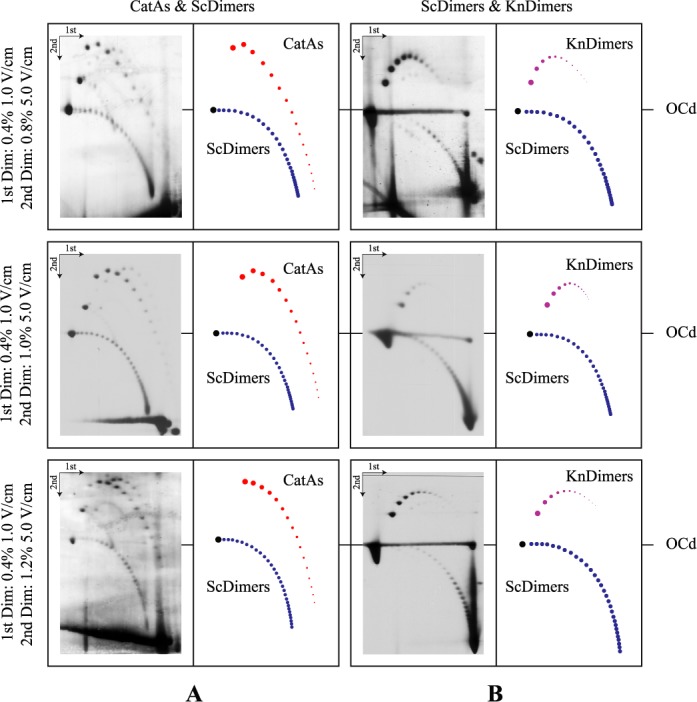
Immunograms of mixed DNA samples enriched for CatAs, ScDimers and KnDimers analyzed by 2D agarose gel electrophoresis where the second dimension was run at the same voltage (5.0 V/cm) in agarose gels of different concentrations. Electrophoresis conditions are detailed to the left and interpretative diagrams are shown to the right. OCd refers to the mobility of dimeric open circles used to align the different immunograms. CatAs are depicted in red, ScDimers in dark blue and KnDimers in pink.

**Figure 5.**
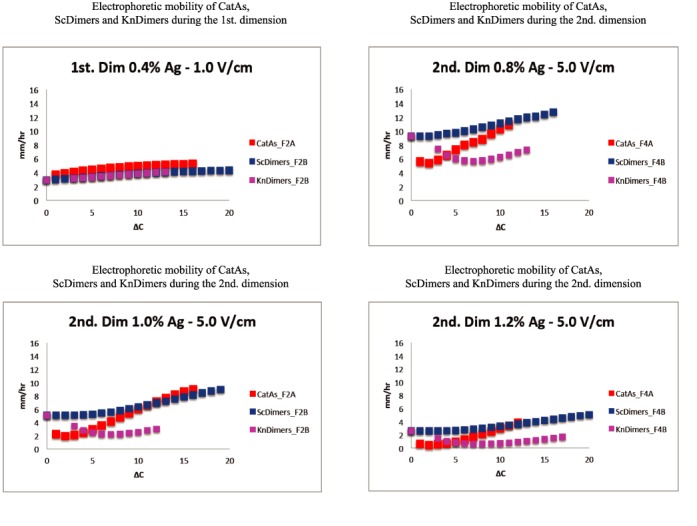
Comparison of the electrophoretic mobility of different topoisomers during the first and second dimension of 2D gels where the latter occurred in gels of different agarose concentrations. Electrophoretic mobility as a function of Δ*C* is expressed in mm/h for the CatAs (depicted in red) shown in Figure 4A and for the ScDimers (depicted in dark blue) and KnDimers (depicted in pink) shown in Figure 4B. The actual numbers are shown in Supplementary Tables S1, S2, S5 and S6).

**Figure 6.**
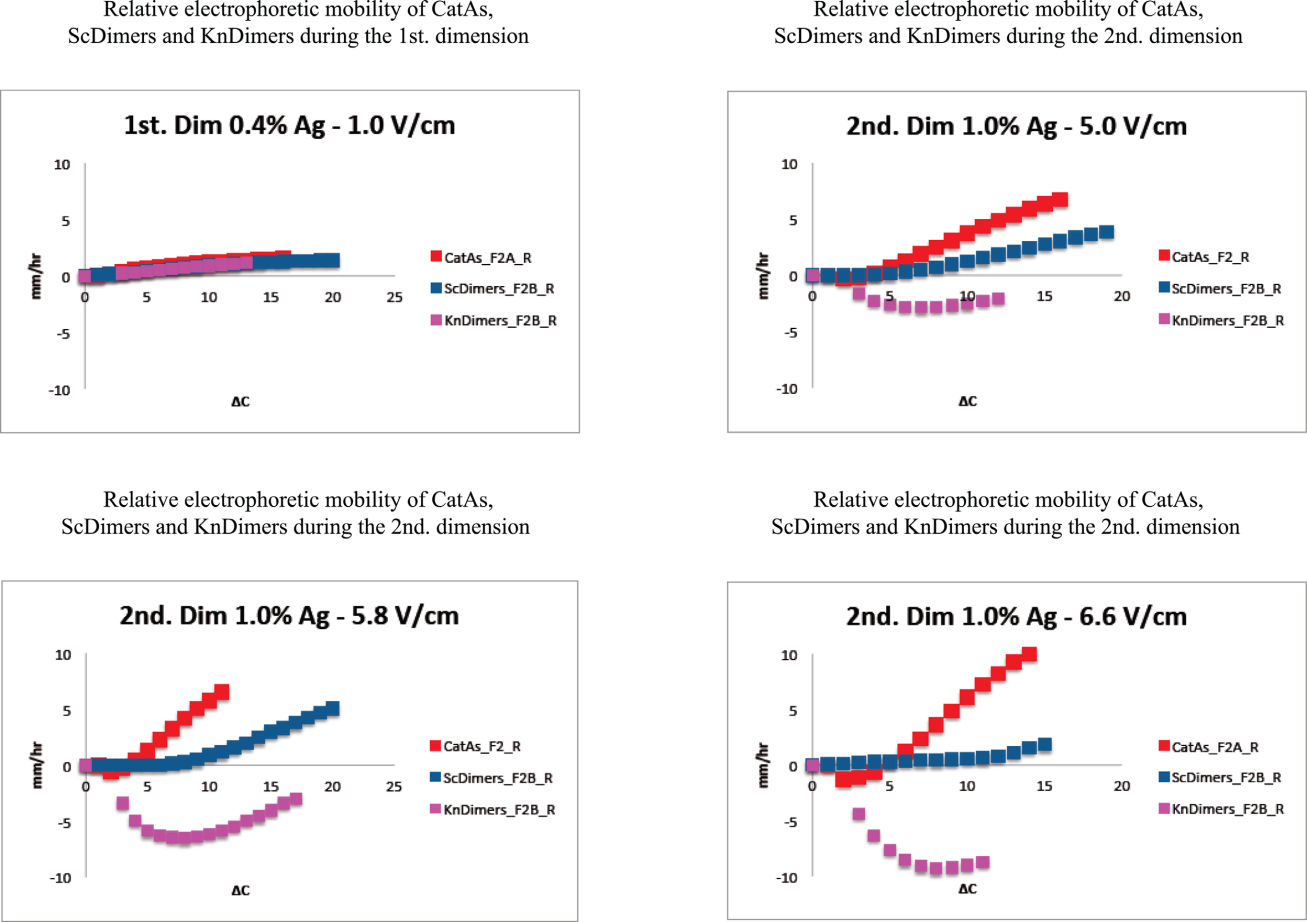
Comparison of the relative electrophoretic mobility of different topoisomers during the first and second dimension of 2D gels where the latter occurred at different voltages. The relative electrophoretic mobility (referred to the mobility of molecules with increasing Δ*C* within each family expressed with respect to that one shown by the simplest member of the family) is expressed in mm/h for the CatAs (depicted in red) shown in Figure 2A and for the ScDimers (depicted in dark blue) and KnDimers (depicted in pink) shown in Figure 2B. The actual numbers are shown in Supplementary Tables S1–S4).

**Figure 7.**
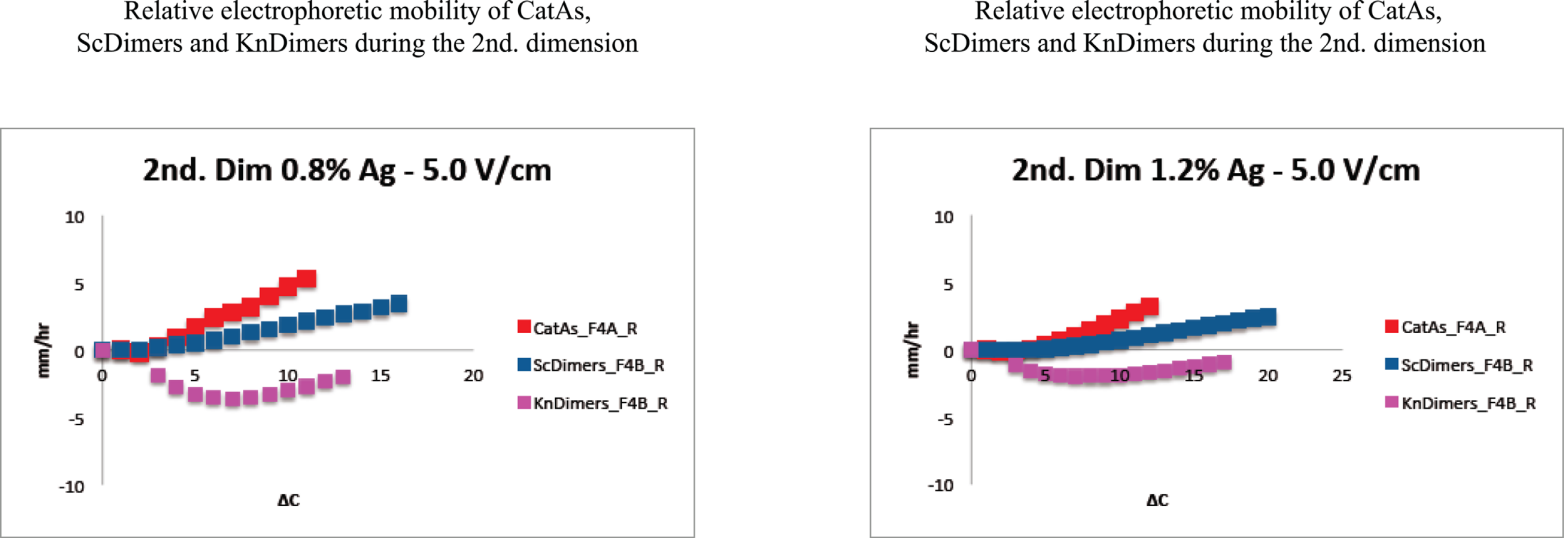
Comparison of the relative electrophoretic mobility of different topoisomers during the second dimension of 2D gels that occurred in gels of different agarose concentrations. The relative electrophoretic mobility (referred to the mobility of molecules with increasing ΔC within each family expressed with respect to that one shown by the simplest member of the family) is expressed in mm/h for the CatAs (depicted in red) shown in Figure 4A and for the ScDimers (depicted in dark blue) and KnDimers (depicted in pink) shown in Figure 4B. The actual numbers are shown in Supplementary Tables S5 and S6).

Two-dimensional (2D) agarose gel electrophoresis is the method of choice to separate the topoisomers of any given circular DNA molecule (1–3). It basically consists in two consecutive runs of electrophoresis performed under different conditions and run at two orthogonal directions (4–8). One of its versions, originally designed by Bell and Byers (4) to separate branched and linear molecules, was subsequently adapted to characterize the replication intermediates (RIs) of any given linear DNA fragment (9). This method is now widely used to map replication origins, termination sites, replication fork stalling and even molecules containing reversed forks (9–13). In this version, the first dimension usually occurs in a low percentage agarose gel (∼0.4%) run at a relatively low voltage (∼1 V/cm). The second dimension occurs perpendicular to the first one, but in a higher percentage agarose gel (∼1%) run at higher voltage (∼5–6 V/cm). It was early noticed that this method could also be successfully applied to analyze different topological forms (14). Supercoiled, catenated and knotted forms are clearly identified as they react in a different way to changes of electrophoretic conditions (15). Several reports demonstrated that in low concentration agarose gels run at low voltage the electrophoretic mobility of different DNA knots and catenanes systematically increases with the average crossing number (ACN), these molecules show in an unperturbed form, since for molecules with the same chain length an increase of ACN necessities increase of overall compaction (16–19). Here, we used low voltage and low agarose concentration during the first dimension and several different conditions during the second dimension of a 2D gel system to examine the roles of voltage and agarose concentration on the electrophoretic mobility of three families of topoisomers with the same mass: supercoiled dimers (ScDimers), nicked-knotted dimers (KnDimers) and monomeric-nicked catenanes (CatAs). Cartoons illustrating these topoisomers are shown in Figure 1. Supercoiled and nicked-knotted monomers are also shown. To facilitate further discussion, we chose to draw representatives of three different families of topoisomers in such a way that they show a similar density of crossings. Comparison of supercoiled, knotted and catenated molecules is not trivial. The essential concepts of DNA topology have been treated in detail in several books and reviews (20–23). In short, in the case of supercoiled DNA molecules their average shape, which is an important factor affecting electrophoretic mobility, depends on the extent of torsional stress acting on the molecules and this in turn depends on how much the linking number of a given supercoiled DNA molecule differs from the linking number of another covalently closed molecule with the same nucleotide sequence. In the case of catenanes analyzed here, molecules were nicked and thus released of torsional stress but they differed in the extent of their catenation number, which informs how many times one double-stranded DNA molecule is topologically linked with the other (24). The differences in catenation number affect the average shape and deformability of catenanes. Finally for the knotted DNA molecules analyzed here, which were also nicked, their shape and deformability depends on the type of formed knots where more complex knots, with higher number of crossings reach a more compact shape than less complex knots (23). As schematically illustrated in Figure 1, ScDimers, KnDimers and CatAs are molecular species of the same mass. For the molecules we chose to examine, the mass of pBR18 monomers is 4383 bp and for dimers it corresponds to 8766 bp. For covalently closed circles (CCCs) the stepwise changes of their electrophoretic mobilities are the consequence of different linking numbers (Lk). For CatAs these stepwise changes are the consequence of different catenation numbers (Ca). For nicked, knotted circles, finally, the stepwise changes in their electrophoretic mobilities are the consequence of different knot types where the decisive contribution comes from different minimal number of crossings characterizing any given knot, although different types of knots with the same minimal crossing number show slightly different electrophoretic mobility (18). Torus type of catenanes and twist type of knots with increasing number of crossings are expected to form regularly spaced bands in arcs of catenated and of knotted DNA molecules. Here we decided to compare the electrophoretic mobility of ScDimers, KnDimers and CatAs based on the empirical observation that for individual members within each family this mobility changes stepwise (Figures 2 and 4). We attributed these changes in mobility to changes in molecular shape that occur stepwise and are results of differences of topological complexity (Δ*C*). Δ*C* is a practical measure that tells how many spaces between DNA bands on a given arc (of supercoiled, of nicked catenated and of nicked knotted molecules) separate a given form from a band formed by simple not-supercoiled DNA circles of the same size. Each point in the graphs shown in Figures 3, 5, 6 and 7 and Supplementary Tables S1–S6 corresponds to the electrophoretic mobility expressed in mm/h of individual spots of each family of topoisomers identified in the immunograms of Figures 2 and 4.

The results obtained indicated that the contribution of increasing Δ*C* to electrophoretic mobility differed for ScDimers, KnDimers and CatAs depending on the conditions of electrophoresis applied.

## MATERIALS AND METHODS

### Bacterial strains, plasmids and culture medium

The *Escherichia coli* strains used in this study were DH5αF′ (supplied by Santiago Rodríguez de Córdoba), parE10 (provided by Ian Grainge) and LZ38 (supplied by Lynn Zechiedrich). Their relevant genotypes are detailed below:

**Table:** 

DH5αF′	F′/*gyrA96*(Nal*r*) *recA1 relA1 endA1 thi-1 hsdR17* (*r_k_*^-^*m_k_^+^*) *glnV44 deoR* Δ(*lacZYA-argF*)*U169*[F80dΔ(*lacZ*)*M15*]
	
parE10	W3110 F- except [*parE10 recA*]
	
LZ38	F-λ (*P80 red114 xis-l cl857*) *zei-723*::Tn*10 parC*K84::*kan*

Competent cells were transformed with monomeric (4383 bp) or dimeric forms (8766 bp) of pBR18, a derivative of pBR322 where the tetracycline resistance promoter was replaced with the poly-linker of pUC18 (12). DH5αF′ and LZ38 cells were grown in LB medium at 37ºC while parE10 cells were grown at 30ºC. In all cases, 75 μg/ml ampicillin was added to the LB medium. Isolation of plasmid DNA was performed as described elsewhere (25,26).

### Cell and DNA treatments

We used norfloxacin to inhibit wild-type DNA gyrase and topoisomerase IV (Topo IV) *in vivo* as described elsewhere (27,28). Drug-resistant mutants allowed us to selectively inhibit DNA gyrase or Topo IV. Cells were treated with 15 or 156 μM Norfloxacin for 15–30 min just before harvest. In all cases the DNA samples analyzed were prepared from separately isolated pools of supercoiled DNAs mixed *in vitro* with nicked forms of knotted or catenated molecules.

To induce single-stranded breaks, DNA was digested with *Nb*.BsmI (New England Biolabs) for 1 h at 8 U/μg of DNA at 50ºC. Reactions were blocked with 100 μg/ml proteinase K (Roche) for 30 min at 37ºC (29).

### 2D agarose gel electrophoresis and Southern transfer

The first dimension was in a 0.4% agarose gel in TBE buffer (89 mM Tris–borate, 2 mM ethylenediaminetetraacetic acid (EDTA)) at 1.0 V/cm at room temperature for 25–30 h. The second dimension was in a 0.8–1.2% agarose gel in TBE buffer that was run perpendicular to the first dimension. The dissolved agarose was poured around the excised agarose lane from the first dimension and electrophoresis was at 5–6.6 V/cm in a 4ºC cold chamber for 8–22 h. Southern transfer was performed as described before (30).

### Non-radioactive hybridization

DNA probes were labeled with digoxigenin using the DIG-High Prime kit (Roche). Membranes were prehybridized in a 20 ml prehybridization solution (2× SSPE (Saline Sodium-Phosphate-EDTA), 0.5% Blotto, 1% SDS, 10% dextran sulfate and 0.5 mg/ml sonicated and denatured salmon sperm DNA) at 65ºC for 4–6 h. Labeled DNA was added and hybridization lasted for 12–16 h. Hybridized membranes were sequentially washed with 2× SSC (Saline Sodium-Citrate) and 0.1% SDS at room temperature for 5 min twice and with 0.1× SSC and 0.1% sodium dodecyl sulfate (SDS) at 68ºC for 15 min twice as well. Detection was performed with an antidigoxigenin-AP conjugate antibody (Roche) and CDP-Star (Perkin Elmer) according to the instructions provided by the manufacturer.

## RESULTS

To obtain broad spectrum of topoisomers of supercoiled dimeric forms of pBR18 we transformed *E. coli* LZ38 cells (31,32) with pBR18 (12). These cells are recA^+^ and accumulate monomeric as well as multimeric forms of the plasmid. In addition they bear a mutation in the parC gene that turns Topo IV resistant to Norfloxacin. This drug inhibits both bacterial type 2 DNA topoisomerases (33), but as in LZ38 cells Topo IV is resistant to Norfloxacin, only DNA gyrase is preferentially affected (34). In this way, it was clearly shown that suboptimal concentrations of the drug turn plasmids poorly supercoiled and monomeric as well as dimeric topoisomers become clearly distinguished (Figure 1A). A similar result could be obtained using chloroquine 2D gels. However, chloroquine would not have helped to separate nicked DNA forming different knots and catenanes since in these molecules the DNA is not under a torsional constraint. The aim of our investigation was to have a good way of separating various topological forms including different types of nicked knots and catenanes.

To obtain DNA preparations enriched in catenanes we transformed *E. coli* DH5∝F’ cells (35) with the same plasmid. These cells are recA^−^ and for this reason few if any multimers form. In addition, DH5∝F’ cells bear a mutation in the gyrA gene that turns DNA gyrase resistant to Norfloxacin. Therefore, in these cells Norfloxacin inhibits only Topo IV leading to the accumulation of catenated duplexes (34,36). Once isolated this DNA was digested with the nicking enzyme *Nb*.BsmI to eliminate supercoiled forms and convert all catenanes into CatAs (Figure 1B).

To obtain knotted dimers, we transformed *E. coli* parE10 cells (37) with pBR18 dimers and the cells were exposed to 15 μM Norfloxacin to partially relax unreplicated supercoiled molecules (34). In this way, supercoiled and knotted dimers could be visualized in the same 2D gel (Figure 1C).

Using the appropriate mixture of DNAs isolated from the different cell types, the electrophoretic mobility of CatAs and ScDimers on one hand, and ScDimers and KnDimers on the other hand, were analyzed in 2D gel systems where the second dimension of the 2D gel system run at different voltages and agarose concentrations (Figures 2–4 and 5). ScDimers were analyzed in two different experiments (Figure 2A and B). Their electrophoretic mobility during the first and second dimensions varied slightly between experiments although the differences were not significant. Therefore, for comparison with the mobility of other topoisomers we used the figures of ScDimers observed in Figure 2B. The electrophoretic mobility of different topoisomers, with their corresponding increase in topological complexity (Δ*C*), expressed in mm/h was compared during the first and the second dimensions and also for the different electrophoretic conditions used during the second dimension (Figures 3, 5, 6 and 7). The actual figures are shown in Supplementary Tables S1–S6.

The results shown in Figures 2–6 confirmed that the electrophoretic mobility of different topoisomers analyzed (ScDimers, KnDimers and CatAs) increased with their expected overall compaction when the molecules were analyzed in low percentage agarose gels run at low voltage (16–19). The electrophoretic mobility for all the families analyzed during the first dimension of the 2D gel system used, run in 0.4% agarose gels at 1 V/cm, systematically increased with the expected increase of the overall compaction of the molecules. However, this was not the case anymore for the electrophoretic runs used during the second dimension. To identify the roles of voltage and agarose concentration on the electrophoretic mobility of the different topoisomers, we run the second dimension of the 2D gel system in 1% agarose gels at three different voltages: 5.0, 5.8 and 6.6 V/cm (Figures 2 and 3) or at the same voltage (5.0 V/cm) but in agarose gels of three different concentrations: 0.8, 1.0 and 1.2% (Figures 3 and 5).

The electrophoretic mobility of different topoisomers of the same mass and Δ*C* = 1 during the first dimension (run in 0.4% agarose gels at 1.0 V/cm) varied between 3.02 mm/h (for ScDimers) and 3.70 mm/h (for CatAs). During the second dimension (run in 1% agarose gels at 5.0, 5.8 and 6.6 V/cm, respectively) these figures were 5.06, 9.49 and 14.09 mm/h for ScDimers and 2.25, 4.59 and 6.38 mm/h for CatAs. Note that the simplest KnDimer, the trefoil knot, has three nodes (18,19). The electrophoretic mobility of all these topoisomers as their Δ*C* increased was significantly different from each other (Figures 2 and 3 and Supplementary Tables S1–S4). These differences became manifested when the relative mobility of the molecules with increasing Δ*C* within each family were expressed with respect to that one shown by the simplest member of the family (Figure 6). Note that during the second dimension for molecules with low Δ*C*, except for ScDimers, the electrophoretic mobility was inversely proportional to their Δ*C* (Figure 6). This behavior was particularly evident for KnDimers where the electrophoretic mobility remained retarded up to molecules with Δ*C* = 8. Surprisingly, for ScDimers during the second dimension, the electrophoretic mobility of topoisomers with increasing Δ*C* remained fairly constant up to Δ*C* = 6, 8 and 14 in gels run at 5.0, 5.8 and 6.6 V/cm, respectively (Figures 2 and 3). On the other hand, during this second dimension, the behavior of CatAs was also curious. Their electrophoretic mobility was retarded for the first 2–4 Δ*C* species but gained mobility very fast with each additional Δ*C* thereafter. This behavior expanded with increasing voltage. Precisely the opposite was observed for KnDimers. In other words, during the second dimension, as voltage increased at a constant agarose concentration (1%) topoisomers with increasing Δ*C* behaved differently. For molecules with relatively low Δ*C* (> 2–4 < 10–15), CatAs gained mobility fast, ScDimers gained very little mobility while KnDimers reduced their mobility (Figures 2 and 3).

The effect of agarose concentration at a constant voltage (5.0 V/cm) during the second dimension, on the other hand, was significantly different (Figures 4 and 5 and Supplementary Tables S5 and S6). The electrophoretic mobility of CatAs and ScDimers with Δ*C* = 1 decreased from 5.60 and 9.25 mm/h (in 0.8% agarose gels) to 0.64 and 2.59 mm/h (in 1.2% agarose gels), respectively. For trefoil KnDimers, their electrophoretic mobility decreased from 7.40 mm/h (in 0.8% agarose gels) to 1.45 mm/h (in 1.2% agarose gels). This behavior applied to almost all topoisomers with increasing Δ*C*. In short, the electrophoretic mobility of all topoisomers studied decreased as agarose concentration increased. This behavior progressively turned the shape of the different curves alike (Figure 5).

## DISCUSSION

As previously mentioned, it was repeatedly shown that the electrophoretic mobility of different DNA knots and catenanes is proportional to the compactness of their unperturbed equilibrium shapes when analyzed in low concentration agarose gels run at low voltage (16–19). The behavior of these topoisomers in high concentration agarose gels run at relatively high voltage received considerable less attention (3). Bell and Byers (4) analyzed X-shaped and linear molecules in 2D gels where the first dimension run in a 0.7% agarose gel at 0.75 V/cm whereas the second dimension run in a 1.5% agarose gel at 2.0 V/cm. An inversion in electrophoresis mobility was observed using a similar 2D gel version for knotted replication intermediates, linear fragments containing an internal knotted bubble (25,38–41). Weber *et al*. used Monte Carlo simulations to investigate the reasons for this reversal of relative order of electrophoretic mobility and concluded that at high electric fields the simulated knotted molecules tend to hang over the gel fibers (42–44). Earlier studies demonstrated that relatively large supercoiled DNA molecules have branched structure (45) and that this may play a significant role in their electrophoretic mobility. Here, we report that the electrophoretic mobility of CatAs, ScDimers and KnDimers with the same molecular mass systematically increased with their progressing compaction during the first dimension of a 2D gel system run in a 0.4% agarose gel at 1.0 V/cm. However, during the second dimension, run in a 0.8–1.2% agarose gel at 5.0–6.6 V/cm knotted and catenated DNA molecules were initially decreasing their electrophoretic mobility with their increasing topological complexity. We propose that when gel pore sizes become smaller than the dimensions of undisturbed equilibrium shapes of analyzed DNA molecules, the deformability and thus the easiness of the molecules to pass through the gel pores becomes the limiting factor of their electrophoretic mobility (46–49). In the case of knotted or catenated DNA molecules, there are two partially opposing effects of increasing topological complexity: (i) A decrease of the spatial extent of the molecules measured by such observables as radius of gyration or the average inverse distance. (ii) An increase of resistance toward further compaction. In low concentration gels where the gel pore sizes are large, the molecules do not need to be squeezed to pass through the gel pores. In such a situation, their electrophoretic mobility simply increases with the complexity of knots and catenanes since more knotted and more catenated DNA molecules are more compact. In gels with higher concentration of agarose, where gel pores are smaller than the average equilibrium size for weakly knotted or catenated DNA molecules, these species experience more difficulties to pass through gel pores than unknotted and uncatenated DNA molecules of the same size that can easily squeeze through the pores. For a simple ring to pass through a narrow pore, it is only needed for two regions to be strongly bent. For knotted and catenated DNA molecules at least four regions need to be strongly bent and this requires more work for every passage through a pore. This finally results in a slower electrophoretic mobility. As knots and catenanes become very complex (more than eight crossings for knots and more than six crossings for catenanes) the molecules increase again their electrophoretic mobility as their knotting and catenation cause strong DNA bending that facilitates passage through the narrow pores in the gel.

In practical terms, for circular molecules of ∼8000 bp, we propose that the best way to distinguish all topoisomers is to run the first dimension in a 0.4% agarose gel at 1.0 V/cm and the second dimension in a 1.0% agarose gel at 6.6 V/cm (Figures 2 and 3).

## SUPPLEMENTARY DATA

Supplementary Data are available at NAR Online.

SUPPLEMENTARY DATA
