# Supplementary material for: Electrophoretic mobility of supercoiled, catenated and knotted DNA molecules
Source: Nucleic Acids Res. 2014 Nov 20;43(4):e24. doi: 10.1093/nar/gku1255 (PMC4344484; doi:10.1093/nar/gku1255)
Supplement: SUPPLEMENTARY DATA [file supp_43_4_e24__index.html]

Electrophoretic mobility of supercoiled, catenated and knotted DNA molecules — Electrophoretic mobility of supercoiled, catenated and knotted DNA molecules — SUPPLEMENTARY DATA 

# Electrophoretic mobility of supercoiled, catenated and knotted DNA molecules

## SUPPLEMENTARY DATA

**Files in this Data Supplement:**

- SUPPLEMENTARY DATA
