# Supplementary material for: Electrophoretic mobility of supercoiled, catenated and knotted DNA molecules
Source: Nucleic Acids Res. 2014 Nov 20;43(4):e24. doi: 10.1093/nar/gku1255 (PMC4344484; doi:10.1093/nar/gku1255)
Supplement: SUPPLEMENTARY DATA [file supp_gku1255_nar-02609-met-f-2014-File009.pdf]

TABLES

Electrophoretic mobility of individual spots of each family of the topoisomers identified in the immunograms of Figures 2 and 4 expressed in mm/hr. The conditions of the run are described on top of each TABLE.

1st. Dimension 0.4% - 1.0 V/cm

# ΔC	CatAs_F2A	ScDimers_F2A	ScDimers_F2B	KnDimers_F2B
0		3,28	2,92	2,92
1	3,70	3,38	3,02	
2	3,90	3,46	3,10	
3	4,08	3,54	3,18	3,16
4	4,26	3,64	3,28	3,26
5	4,40	3,74	3,38	3,34
6	4,52	3,84	3,48	3,44
7	4,64	3,94	3,58	3,54
8	4,74	4,04	3,70	3,62
9	4,84	4,12	3,78	3,72
10	4,90	4,20	3,86	3,82
11	4,98	4,28	3,94	3,92
12	5,06	4,34	4,00	4,02
13	5,10	4,40	4,06	4,08
14	5,16	4,46	4,12	
15	5,20	4,48	4,16	
16	5,26	4,54	4,20	
17		4,58	4,24	
18		4,62	4,28	
19			4,30	
20			4,32	

TABLE 1

2nd. Dimension **1% - 5.0 V/cm**

# ΔC	CatAs_F2A	ScDimers_F2A	ScDimers_F2B	KnDimers_F2B
0		5,50	5,00	5,00
1	2,25	5,50	5,00	
2	1,95	5,50	5,00	
3	2,10	5,50	5,03	3,38
4	2,45	5,50	5,06	2,78
5	2,95	5,60	5,22	2,41
6	3,55	5,65	5,41	2,25
7	4,15	5,80	5,59	2,22
8	4,75	5,90	5,78	2,25
9	5,35	6,15	6,06	2,41
10	6,00	6,35	6,31	2,53
11	6,60	6,60	6,59	2,78
12	7,15	6,90	6,91	3,00
13	7,65	7,15	7,19	
14	8,20	7,40	7,47	
15	8,65	7,65	7,78	
16	9,00	7,90	8,03	
17		8,15	8,19	
18		8,35	8,63	
19			8,91	

TABLE 2

2nd. Dimension 1% - 5.8 V/cm

# ΔC	CatAs_F2A	ScDimers_F2A	ScDimers_F2B	KnDimers_F2B
0		10,55	9,49	9,49
1	4,59	10,55	9,49	
2	4,05	10,59	9,49	
3	4,36	10,64	9,49	6,11
4	5,00	10,64	9,49	4,55
5	5,90	10,64	9,49	3,64
6	6,90	10,68	9,49	3,25
7	7,86	10,68	9,63	3,06
8	8,77	10,73	9,75	2,99
9	9,64	10,77	10,01	3,12
10	10,36	10,91	10,40	3,38
11	11,09	11,14	10,66	3,64
12		11,41	11,05	4,03
13		11,73	11,44	4,55
14		12,14	11,96	4,94
15		12,55	12,48	5,46
16		12,95	12,80	6,11
17		13,41	13,26	6,50
18		13,86	13,71	
19		14,27	14,17	
20		14,72	14,56	

TABLE 3

2nd. Dimension **1% - 6.6 V/cm**

# ΔC	CatAs_F2A	ScDimers_F2A	ScDimers_F2B	KnDimers_F2B
0		14,19	14,09	14,09
1	6,38	14,19	14,09	
2	5,13	14,25	14,16	
3	5,25	14,38	14,23	9,74
4	5,75	14,44	14,31	7,74
5	6,69	14,50	14,38	6,49
6	7,63	14,56	14,38	5,53
7	8,75	14,69	14,46	5,02
8	10,00	14,75	14,53	4,79
9	11,25	14,81	14,53	4,87
10	12,50	14,88	14,60	5,09
11	13,63	14,94	14,68	5,38
12	14,63	15,00	14,75	
13	15,63	15,06	14,90	
14	16,38		15,19	
15			15,64	
16			15,93	

TABLE 4

2nd. Dimension **0.8% - 5.0 V/cm**

# ΔC	CatAs_F4A	ScDimers_F4A	ScDimers_F4B	KnDimers_F4B
0		9,40	9,25	9,25
1	5,60	9,40	9,25	
2	5,40	9,40	9,25	
3	5,85	9,50	9,45	7,40
4	6,55	9,40	9,65	6,50
5	7,30	9,50	9,75	6,00
6	8,00	9,65	10,0	5,75
7	8,40	9,80	10,25	5,65
8	8,80	10,10	10,55	5,75
9	9,60	10,40	10,80	5,95
10	10,30	10,60	11,10	6,25
11	10,90	10,90	11,40	6,55
12		11,15	11,70	6,95
13		11,50	11,95	7,30
14		11,80	12,10	
15		12,05	12,40	
16			12,70	

TABLE 5

2nd. Dimension **1.2% - 5.0 V/cm**

# ΔC	CatAs_F4A	ScDimers_F4A	ScDimers_F4B	KnDimers_F4B
0		3,02	2,59	2,59
1	0,64	3,02	2,59	
2	0,45	3,02	2,59	
3	0,57	3,05	2,59	1,45
4	0,68	3,11	2,61	1,02
5	0,95	3,18	2,64	0,82
6	1,27	3,27	2,75	0,68
7	1,68	3,41	2,86	0,61
8	2,09	3,59	2,98	0,64
9	2,50	3,77	3,14	0,66
10	2,95	3,95	3,30	0,70
11	3,41	4,34	3,48	0,80
12	3,86	4,73	3,64	0,91
13		4,91	3,82	1,05
14		5,59	4,00	1,18
15			4,18	1,36
16			4,36	1,50
17			4,55	1,68
18			4,73	
19			4,91	
20			5,05	

TABLE 6
